# Control tissue in brain banking: the importance of thorough neuropathological assessment

**DOI:** 10.1007/s00702-015-1376-6

**Published:** 2015-02-12

**Authors:** M. Nolan, C. Troakes, A. King, I. Bodi, S. Al-Sarraj

**Affiliations:** 1 Department of Basic and Clinical Neuroscience, Institute of Psychiatry, Psychology and Neuroscience, Kings College London, London, UK; 2 Department of Clinical Neuropathology, Kings College NHS Foundation Trust, London, UK; 3 Department of Clinical Neuropathology, 1st Floor, Academic Neuroscience Centre, King’s College Hospital, Denmark Hill, London, SE5 9RS UK

**Keywords:** Brain banking, Control tissue, Neuropathology, Brain donation, Histology, Tissue protocols

## Abstract

Historically, control brain tissue was classified as such mainly by clinical history, and underwent limited neuropathological analysis. Significant progress has been made in recent years with the collection of more extensive clinical information and more specific classifications of neurodegenerative disease, aided by advances in histological processing and increasingly sensitive detection methods. We hypothesised that this may have resulted in certain pathologies previously going unidentified, due to insufficient block sampling and an inadequate range of stains, resulting in the disease not being recognised. We therefore investigated the significance of changes to our own protocols for examining control brain tissue before and after 2007. Control cases that were originally assessed before 2007 were re-assessed using our current staining protocol and antibodies, and compared with age-matched cases post-2007. We found that almost all cases that were originally described as neuropathologically normal displayed some level of pathology after re-analysis, with four cases displaying what we have termed ‘major’ pathology that previously went unidentified, emphasising on a small scale the importance of accurate neuropathological analysis of control tissue, and highlighting the inherent difficulty of traditionally classifying tissue simply as ‘disease’ or ‘control’. We hope our findings will stimulate debate within the brain banking community, with the eventual aim being standardisation of protocols for assessing controls across brain banks.

## Introduction

Cases of neurodegenerative diseases such as Alzheimer’s disease are expected to double every 20 years globally until 2040, consistent with an ageing population (Mayeux and Stern 2012). Over the last 30 years, brain banks have become an important resource in the study of both neurodegenerative and neuropsychiatric illness, providing researchers with well-defined collections of both frozen and formalin-fixed tissue in conjunction with accurate clinical information and neurological staging.

The use of control tissue for comparative studies is a necessity. However, high-quality control tissue remains relatively scarce, and researchers often cite the lack of high-quality tissue as impeding research efforts (Samarasekera et al. 2013). Factors contributing to the difficulty in procuring such tissue include a general decline in autopsy rates (Burton and Underwood 2007) and recent organ retention scandals in several countries, including the Alder Hey scandal in the UK (Burton and Wells 2001; McGuone and Kay 2004; Redfern et al. 2001; Sheach Leith 2007) which have damaged the public’s perception of such services. Additionally, awareness of brain donation amongst potential donors is low (Eatough et al. 2012; Kuhta et al. 2011) and recruitment of control donors is significantly lower than disease patients (Bell et al. 2008). It is therefore crucial that donated tissue described as clinically ‘normal’ is appropriately neuropathologically investigated to maximise its potential use.

Several neuropathological methods of staging exist for various neurodegenerative diseases (Alafuzoff et al. 2006; Braak and Braak 1991; Braak et al. 2003; Josephs et al. 2014) and detailed methods of processing FFPE and frozen tissue for brain banking have been described (Vonsattel et al. 1995; Vonsattel et al. 2008; Waldvogel et al. 2006). However, there has so far been no attempt to standardise staining protocols for control tissue. Deciding what is and what is not suitable for designation as control tissue is often inconsistent, compounding the difficulty in providing high-quality, variable-matched tissue to researchers—who often require different definitions of control tissue based on the clinical/pathological nature of their study. Here, we describe and investigate our own protocols for handling control tissue, with the aim of determining the correct balance of thorough investigation with minimisation of workload and cost.

## Methods

We sought to identify differences in pathology revealed using the neuropathological methods employed in the MRC London Neurodegenerative Diseases Brain Bank before and after 2007, when a new, more extensive in-house protocol (Table 1) for assessing control tissue was introduced, including the sampling of a wider range of brain areas and the use of ‘new’ antibodies such as p62 and TDP-43 which were not commercially available before 2007. This protocol is based upon the recommendations by the BrainNet Europe consortium (Al-Sarraj 2008), to include amygdala, occipital lobe, middle frontal gyrus, superior frontal gyrus, superior temporal gyrus, hippocampus, midbrain and parietal lobe (see Table 1). In both groups, only cases that were originally described as clinically control (i.e. no history of neurological disease or cognitive decline) and above 70 years of age were included. Additionally, cases in the pre-2007 group were recorded as having no significant pathology at the time of the original investigation. All cases were originally donated to the MRC London Neurodegenerative Disease Brain Bank.

**Table 1:** Staining protocol for control cases

Fixed region taken	Stains performed
Amygdala (including entorhinal cortex)	H&E, α-synuclein, Tau, TDP-43
Occipital lobe at the level of the calcarine fissure	H&E, β-amyloid, Tau
Middle frontal gyrus and white matter	H&E, β-amyloid, Tau
Superior frontal gyrus including anterior cingulate gyrus	H&E, α-synuclein
Hippocampus (including transentorhinal cortex)	H&E, Bielschowsky, Tau, TDP-43
Midbrain	H&E, α-synuclein, Tau
Parietal lobe and white matter	H&E, Tau
Superior temporal gyrus	H&E, β-amyloid, Tau, TDP-43

The staining protocol (right) was developed in house and since 2007 is performed as standard for all cases clinically assessed as control

### Histology

7-µm sections were cut from formalin-fixed paraffin-embedded (FFPE) tissue. Haematoxylin and eosin staining and modified Bielschowsky staining were performed on 7- and 14-µm sections, respectively, according to standard protocols.

### Immunohistochemistry

The following antibodies were used for immunohistochemistry: anti-tau (clone AT8, 1:500, Thermo Scientific, Massachusetts, USA), anti-phosphorylated-TDP-43 (1:1500, Cosmo Bio, Tokyo, Japan), anti-β-amyloid (clone A4, 1:6000, Covance, New Jersey, USA), anti-α-synuclein (1:500, BD Transduction Laboratories, Kentucky, USA) and anti-p62 (1:100, BD Transduction Laboratories, Kentucky, USA). 7-µm paraffin sections were dewaxed, dehydrated and stained using the listed antibodies on an automated immunohistochemistry platform (Leica BONDMAX^©^, Leica Biosystems). Antibody–antigen interactions were located in the sections using a Polymer-based detection kit (Novocastra^©^ Bond Polymer Refine Detection, Leica Biosystems, Switzerland) and visualised using 3,3′-diaminobenzidine tetrahydrochloride as a chromogen. Nuclei were counterstained using haematoxylin. All cases were assessed by a Consultant Neuropathologist.

#### Post-2007

No additional sampling or staining was conducted for cases that were processed after 2007 (*n* = 39). Each case was retrospectively categorised according to the significance of its previously identified pathology for comparative purposes. Cases which showed no discernible pathology were designated ‘no pathology’. Cases which showed one or more of the following pathologies were designated ‘minor pathology’: mild amyloid angiopathy (i.e. only very occasional leptomeningeal vessels staining for β-amyloid within their walls), mild cerebrovascular disease and very localised tau deposition consistent with modified Braak (BNE) stage I and II (Braak et al. 2006). Cases which showed any of the following pathologies were designated ‘major pathology’: any TDP-43 pathology, any α-synuclein positive Lewy body pathology (including Lewy neurites), significant amyloid angiopathy (at least moderate numbers of leptomeningeal parenchymal vessels staining for β-amyloid within their walls), significant cerebrovascular disease (infarcts greater than 0.5 cm in diameter and/or at least moderate white matter rarefaction with moderate perivascular spacing and moderate thickening of vessel walls) (Deramecourt et al. 2012), argyrophilic grain disease and widespread tau deposition consistent with at least modified Braak (BNE) stage III–VI (Braak et al. 2006).

#### Pre-2007

7-µm FFPE sections were cut from the blocks used in the original investigation and stained using automated immunohistochemistry and the described protocol (Table 1). Pre-2007 cases (*n* = 17) were at the time of original investigation clinically and pathologically diagnosed as control tissue. Blocks from the original investigation were used wherever possible, however in all cases it was necessary to use blocks processed from recently archived wet tissue for some areas; for these an additional p62 stain was performed on blocks requiring α-synuclein to account for possible decreases in immunoreactivity resulting from prolonged formalin fixation (Pikkarainen et al. 2010). These cases were then also categorised according to pathology severity detailed above in the post-2007 group.

## Results

### Post-2007

Despite all cases being classified as control, no cases that were described clinically as neurologically and cognitively normal were completely devoid of pathology. In 35 % of cases this was minor, i.e. tau pathology in keeping with BNE stage I–II, however 65 % displayed at least one major pathology, such as tau pathology in keeping with BNE stage III or TDP-43 pathology of varying degrees. Several cases displayed limbic stage Lewy body disease (see Table 2 for full diagnosis). One interesting case in this group displayed pathology consistent with basal ganglia predominant fronto-temporal lobar degeneration and TDP-43 proteinopathy (FTLD-TDP). However, when the patients’ medical history was re-examined, a broad clinical diagnosis of neurodegenerative disease was discovered and the case was therefore removed from the study.

**Table 2:** Post-2007 cases show differing levels of pathology despite clinical assessment as control

Year	Sex	Age	Agonal state	Neuropathological diagnosis
Minor pathology				
2013	F	86	Coronary artery disease	AD changes, BNE stage II, mild amyloid angiopathy
2013	F	81	Epilepsy	AD changes, BNE stage I, hippocampal sclerosis
2013	M	90	Myocardial Infarction	AD changes, BNE stage +, mild focal amyloid angiopathy
2012	M	91	Stroke	AD changes, BNE stage II
2012	F	85	Breast cancer	AD changes, BNE stage II
2011	M	96	Not available	AD changes, BNE stage II
2011	F	79	Not available	AD changes, BNE stage II
2011	M	74	Respiration failure; cancer	AD changes, BNE stage I
2011	M	97	Bronchopneumonia	AD changes, BNE stage II
2011	F	93	Carcinomatosis	AD changes, BNE stage I, mild amyloid angiopathy
2010	F	84	Metastatic breast cancer	AD changes, BNE stage I, mild amyloid angiopathy
2009	F	84	Not available	AD changes, BNE stage II
2011	F	84	Myocardial infarction	AD changes, BNE stage II
Major pathology				
2013	M	78	Pancreatic cancer	Moderate to severe (diffuse) Cerebrovascular disease
2013	M	81	Prostate cancer	Limbic stage of Alzheimer’s disease, consistent with BNE stage III, mild amyloid angiopathy, hippocampal sclerosis
2013	M	80	Coronary stenosis	AD changes, BNE stage III, mild to moderate small vessel disease
2013^a^	M	81	Pulmonary thromboembolism	AD changes, BNE stage IV, brainstem predominant Lewy body disease, mild amyloid angiopathy
2013^a^	M	84	Colorectal cancer	Occasional Lewy bodies and α-synuclein positive inclusions in the substantia nigra and locus coeruleus. TDP-43 inclusions in the amygdala, hippocampus and parahippocampal gyrus
2013^a^	F	78	NSTEMI	AD changes, BNE stage II, small vessel cerebrovascular disease; focal amyloid angiopathy, limbic TDP-43 pathology
2013	M	85	Heart failure	AD changes, BNE stage III, very focal amyloid angiopathy
2012	M	87	Lung cancer	AD changes, BNE stage III, widespread amyloid angiopathy
2012	F	100	Cerebral ischaemia	AGD, BNE stage III, mild amyloid angiopathy
2012	F	88	Not available	AD changes, BNE stage III
2012	M	79	Pulmonary fibrosis	AD changes, BNE stage III
2012	F	92	Skin cancer; diverticulitis	AD changes, BNE stage III
2011	M	93	Perforated bowel	AD changes, BNE stage IV, mild small vessel disease
2011	F	92	Peritonitis	AD changes, BNE stage IV
2011	F	86	Aspiration pneumonia; stroke; ischaemic heart disease	AD changes, BNE stage III
2011	F	86	Lung cancer	AD changes, BNE stage III
2010	F	84	Lung cancer	AD changes, BNE stage III
2011^a^	M	74	Resp failure; exacerbation of COPD	Mild neocortical Lewy body disease, intermediate probability of AD changes, BNE stage III
2009	M	80	Cancer	AD changes, BNE stage III, mild amyloid angiopathy
2009^a^	F	99	Bronchopneumonia	Posterior type Alzheimer’s disease intermediate stage (NIA-Reagan); limbic TDP-43 positive structures
2012	M	78	Chest infection	AD changes, BNE stage III
2011^a^	M	88	Bronchopneumonia	AD changes, BNE stage IV, amyloid angiopathy and limbic TDP-43 pathology
2008^a^	M	86	Not available	AD changes, BNE stage III, AGD and limbic Lewy body disease
2012^a^	F	90	Transient ischaemic attack	AD changes, BNE stage II, TDP-43 pathology in hippocampus and amygdala, small blood vessel disease

*AD* Alzheimer’s disease, *BNE* BrainNet Europe, *BDR* brains for dementia research, *COPD* chronic obstructive pulmonary disease, *NSTEMI* non-ST elevated myocardial infarction, *AGD* argyrophilic grain disease

^a^Several cases displayed TDP-43 pathology, as well as Lewy body disease. Interestingly, not a single case processed after 2007 displayed no discernible pathology

### Pre-2007

**Fig. 1:**
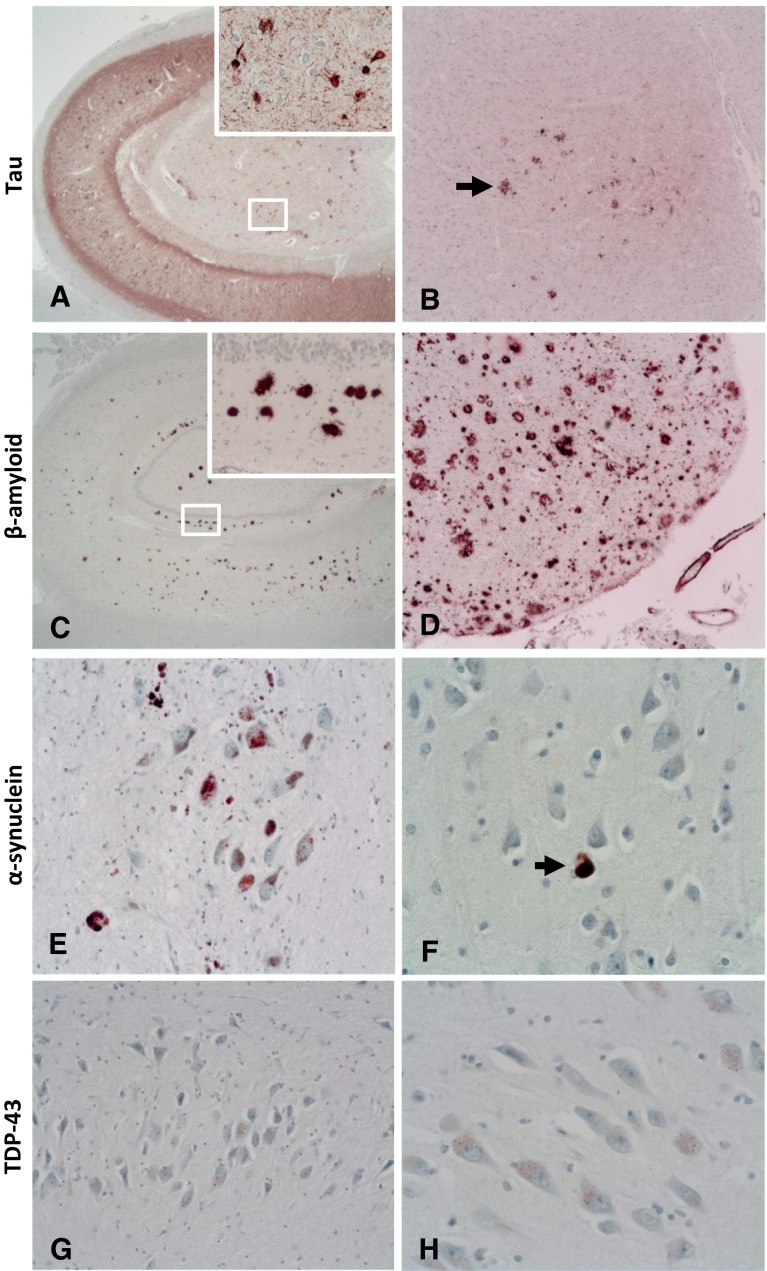
Demonstration of different protein aggregates across brain regions in a case originally investigated in 1993. These aggregates were only identified after re-investigation using our current control tissue protocol. Re-investigation revealed extensive tau deposition consistent with BNE stage V and the presence of Lewy Bodies in the neocortex. **a** Example of tau (AT8) deposition in the hippocampus. **b** Example of tau deposition in the parietal cortex, *arrow* focal tau staining. **c** Example of β-amyloid (A4) deposition in the hippocampus. **d** Example of β-amyloid deposition in the parietal cortex. **e** Example of Lewy body formation highlighted by α-synuclein staining in the midbrain. **f** *Arrow* example of Lewy body formation highlighted by α-synuclein staining in the frontal cortex. **g**, **h** TDP43 reveals only non-specific labelling in a few neurons with granulovacuolar degeneration in the hippocampus. Magnification: **a** ×30, *inset* ×250, **b** ×100, **c** ×50, *inset* ×250, **d** ×100, **e** ×400, **f** ×400, **g** ×200, **h** ×400

All re-analysed clinically control cases except one displayed some level of pathology (Table 3) with 24 % showing pathology deemed major. Notably, one case (aged 92 years) showed severe Alzheimer-type changes, including plaques and tangles consistent with Alzheimer’s disease BNE stage V, limbic subtype of diffuse Lewy body disease and significant amyloid angiopathy (see Table 4) (Fig. 1). Additional α-synuclein staining of temporal, superior and parietal cortices was carried out on this case to confirm Lewy Body staging, after which the diagnosis was altered to diffuse Lewy body disease of mild neocortical subtype. When first investigated (albeit using limited staining/sampling), this case was neuropathologically diagnosed as simply having ‘changes consistent with ageing’, highlighting the increased range and depth of neuropathology being performed now in comparison with 20 years ago.

**Table 3:** Pathology summary table

Post-2007		Pre-2007	
No pathology			
0	0 %	1	6 %
Minor pathology			
13	35 %	12	70 %
Major pathology			
24	65 %	4	24 %
Total			
37	100 %	17	100 %

All cases before 2007, except one, displayed some form of pathology after re-investigation using our current protocol

**Table 4:** Pre-2007 cases that were re-analysed display previously unidentified pathologies

Year	Sex	Age	Agonal state	Original stains performed	Original neuropathology diagnosis	New neuropathology diagnosis
No pathology						
1990^a^	M	70	Bronchopneumonia	H&E, LFB, GFAP, Glees, A4, Gallyas	Normal adult brain	Normal brain
Minor pathology						
1989	F	71	Bronchopneumonia	None	Normal adult brain	AD changes, BNE stage +
1991	F	80	Pulmonary embolism	H&E, Bielschowsky	Normal adult brain	AD changes, BNE stage +
1993	F	79	Ischaemic heart disease	H&E, Bielschowsky	Normal adult brain	AD changes, BNE stage I
1993	M	78	Left ventricular failure	H&E, Bielschowsky	Normal adult brain	AD changes, BNE stage I, mild amyloid angiopathy
1993	M	74	Haemopericardium	H&E, Bielschowsky	Normal adult brain	AD changes, BNE stage I
1998	F	71	Haemothorax	H&E, LFB/N	Normal adult brain	AD changes, BNE stage I
2001	M	71	Colon cancer	H&E, LFB/N, tau, ubiquitin, a-synuclein, Gallyas, GFAP, 12F10	Normal adult brain	AD changes, BNE stage I
2002	F	79	Carcinoma of lung	H&E, tau, LFB	Normal adult brain	AD changes, BNE stage II
2002	M	78	GI haemorrhage	H&E, Bielschowsky	Normal adult brain	AD changes, BNE stage II
2004	M	80	Cerebral amyloid angiopathy	H&E, A4, Bielschowsky, tau	Normal adult brain	AD changes, BNE stage II
2004	F	89	Unknown	H&E, LFB	Normal adult brain	AD changes, BNE stage II, mild amyloid angiopathy
2006	F	82	Cancer	Unknown	Normal adult brain	AD changes, BNE stage I
Major pathology						
1990	F	80	Post-operative haemorrhage	H&E, LFB, GFAP, Glees, A4, Gallyas	Normal adult brain	AD changes, BNE stage III, mild small vessel disease
1992	M	70	Ischaemic heart disease	H&E, Bielschowsky, Congo red, GFAP	Normal adult brain	AD changes, BNE stage III, marked amyloid angiopathy
1993	F	89	Pulmonary emboli	None	Normal adult brain	AD changes, BNE stage III, amyloid angiopathy
1993^b^	F	92	Myocardial infarction	H&E, Bielschowsky	Consistent with ageing	AD changes: BNE stage V, mild neocortical stage of diffuse Lewy body disease, amyloid angiopathy

*AD* Alzheimer’s disease, *CAA* cerebral amyloid angiopathy, *DLBD* diffuse Lewy body disease, *SVD* small vessel disease

^a^Only one case that was previously described as neuropathologically ‘normal’ was also found to be so after re-analysis

^b^Notably, one case from 1993 displays significant pathology which was largely unidentified when originally investigated

## Discussion

The pathological diagnosis of neurodegenerative diseases now rests heavily on the use and interpretation of a range of immunohistochemical stains. As the use of these sensitive stains has developed, there has been a realisation that, to make a sensible diagnosis in the background of clinical information, there is a need to define a benchmark as to what is an expected “normal range” of staining patterns for these markers. In other words, there has become a greater need as to how one defines “control brain tissue”. It is well recognised, for example, that during ageing the brain may develop some tau pathology, without the patient being cognitively impaired (Bennett et al. 2012; Crary et al. 2014; Mitchell et al. 2002; Price et al. 2009). The difficulty comes therefore when one wants to compare some aspects of a brain (with, for example, Alzheimer’s disease) pathology with an age-matched “control” which also may have some tau pathology albeit often mild. The purpose of this study was to determine whether adopting a more thorough blocking and staining protocol for “control” cases in our laboratory has made a difference in detecting otherwise unexpected pathology, and if so the likely significance of this.

After the more recent and comprehensive sampling and staining protocols, we found that out of 17 “control” cases originally assessed before 2007, all but one displayed some level of pathology which were not reported originally; including four cases which showed what we termed “major” pathology and 12 with “minor” pathology. These results are in direct contrast to the original neuropathological descriptions in which only limited sampling and stains were conducted. The results therefore showed that a comprehensive blocking and staining protocol was required to determine the extent of any pathological features in so-called “control” cases. Since “control” brain tissue is the most requested single condition from brain banks, it is certainly important to know what pathological features (if any) each such case shows.

The study of the alteration of the biochemical and molecular components of proteins in specific neuro-anatomical areas is becoming more important and therefore the necessity of knowing the pathological features of corresponding areas in “control” tissue has assumed greater importance. For example, researchers interested in the study of tau, α-synuclein or TDP-43 pathologies in the hippocampus would require knowing to what extent such pathology presents in this region in “control” cases. Our study demonstrated that involvement of transrhinal and entorhinal cortex and hippocampus with tau pathology is in fact frequent in control brains from aged patients and a few even have additional TDP-43 pathology. The importance of this issue becomes more obvious when further sampling and staining identified major pathological diagnosis such as cases of Diffuse Lewy body dementia (DLBD) and amyloid angiopathy in our study.

Another point we found is that acquiring a more detailed clinical history is as important as conducting extra staining and sampling of the brain tissue. One of the cases referred to our brain bank as “control” was found to have extensive TDP43 pathology consistent with FTLD-TDP after more sampling and staining was conducted which would not have been detected without using the more comprehensive recent protocol. However, after requesting further clinical information, there was an indication of cognitive decline and behavioural abnormalities, although these were not clinically characterised before death and brain donation. The importance of obtaining good clinical details and patient assessment before death becomes clearer in trying to answer the question of what constitutes a “control” and what we should consider “pathology within the normal ageing process”. While all the cases used above are age matched over 70 years, it is widely acknowledged that a certain level of tau pathology is often present with ageing, such as in the case of Primary age-related tauopathy (PART) (Crary et al. 2014). However, the precise correlation between age, naturally accepted level of pathology and suitability as control tissue is debatable. We suggest that it may be better to view the pathology of “control” brains from aged donors as a spectrum of pathologies rather than a discrete grouping and to be clearly matched with clinical information before death—particularly assessment of Mini mental state examination (MMSE), especially given the debate surrounding whether patients with mild pathology are essential in the early stage of disease even without clinical symptoms. These issues raise the question of the actual usefulness of accepting “ad-hoc” brain donation without prior clinical assessment as has been our practice for some time.

Lastly, our approach has obvious limitations. For example, the neuropathological diagnosis of Alzheimer’s Disease is based around tau pathology and BNE staging. We only considered to a lesser degree the compounding effect of co-existing pathologies such as vascular pathology which would require further consideration. Our sampling protocol is based on broad sampling of brain tissue and a number of immunohistochemical stains; however, it may not be applicable in all instances and all brain banks. The issue of how many regions to sample and how many immunohistochemical tests to request on clinically defined control tissue is to a certain degree a matter of pathologist preference and often a matter of cost for individual brain banks. Our diagnostic protocol did not until recently provide for the investigation of the earliest pathological manifestation of possible Lewy body disease in the medulla and pons, but adding further stains to an ever-increasing list does present potential economic problems.

## References

[CR1] Alafuzoff I, Pikkarainen M, Al-Sarraj S, Arzberger T, Bell J, Bodi I, Kretzschmar H (2006). Interlaboratory comparison of assessments of Alzheimer disease-related lesions: a study of the BrainNet Europe Consortium. J Neuropathol Exp Neurol.

[CR2] Al-Sarraj S (2008) Neurodegenerative disease protocols. BrainNet Europe Consortium, 1–4

[CR3] Bell JE, Alafuzoff I, Al-Sarraj S, Arzberger T, Bogdanovic N, Budka H, Kretzschmar H (2008). Management of a twenty-first century brain bank: experience in the BrainNet Europe consortium. Acta Neuropathol.

[CR4] Bennett DA, Wilson RS, Boyle PA, Buchman AS, Schneider JA (2012). Relation of neuropathology to cognition in persons without cognitive impairment. Ann Neurol.

[CR5] Braak H, Braak E (1991). Neuropathological stageing of Alzheimer-related changes. Acta Neuropathol.

[CR6] Braak H, Del Tredici K, Rub U, de Vos RA, Jansen Steur EN, Braak E (2003). Staging of brain pathology related to sporadic Parkinson’s disease. Neurobiol Aging.

[CR7] Braak H, Alafuzoff I, Arzberger T, Kretzschmar H, Del Tredici K (2006). Staging of Alzheimer disease-associated neurofibrillary pathology using paraffin sections and immunocytochemistry. Acta Neuropathol.

[CR8] Burton JL, Underwood J (2007). Clinical, educational, and epidemiological value of autopsy. Lancet.

[CR9] Burton JL, Wells M (2001). The Alder Hey affair: implications for pathology practice. J Clin Pathol.

[CR10] Crary JF, Trojanowski JQ, Schneider JA, Abisambra JF, Abner EL, Alafuzoff I, Nelson PT (2014). Primary age-related tauopathy (PART): a common pathology associated with human aging. Acta Neuropathol.

[CR11] Deramecourt V, Slade JY, Oakley AE, Perry RH, Ince PG, Maurage CA, Kalaria RN (2012). Staging and natural history of cerebrovascular pathology in dementia. Neurology.

[CR12] Eatough V, Shaw K, Lees A (2012). Banking on brains: insights of brain donor relatives and friends from an experiential perspective. Psychol Health.

[CR13] Josephs KA, Murray ME, Whitwell JL, Parisi JE, Petrucelli L, Jack CR, Dickson DW (2014). Staging TDP-43 pathology in Alzheimer’s disease. Acta Neuropathol.

[CR14] Kuhta T, Zadikoff C, Simuni T, Martel A, Williams K, Videnovic A (2011). Brain donation–what do patients with movement disorders know and how do they feel about it?. Parkinsonism Relat Disord.

[CR15] Mayeux R, Stern Y (2012). Epidemiology of Alzheimer disease. Cold Spring Harb Perspect Med.

[CR16] McGuone D, Kay EW (2004). The impact of the organ retention controversy on the practice of hospital necropsy: a four year audit. J Clin Pathol.

[CR17] Mitchell TW, Mufson EJ, Schneider JA, Cochran EJ, Nissanov J, Han LY, Arnold SE (2002). Parahippocampal tau pathology in healthy aging, mild cognitive impairment, and early Alzheimer’s disease. Ann Neurol.

[CR18] Pikkarainen M, Martikainen P, Alafuzoff I (2010). The effect of prolonged fixation time on immunohistochemical staining of common neurodegenerative disease markers. J Neuropathol Exp Neurol.

[CR19] Price JL, McKeel DW, Buckles VD, Roe CM, Xiong C, Grundman M, Morris JC (2009). Neuropathology of nondemented aging: presumptive evidence for preclinical Alzheimer disease. Neurobiol Aging.

[CR20] Redfern M, Keeling JW, Powell E (2001) Liverpool Children’s Hospital inquiry report. London

[CR21] Samarasekera N, Al-Shahi Salman R, Huitinga I, Klioueva N, McLean CA, Kretzschmar H, Ironside JW (2013). Brain banking for neurological disorders. Lancet Neurol.

[CR22] Sheach Leith VM (2007). Consent and nothing but consent? The organ retention scandal. Sociol Health Illn.

[CR23] Vonsattel JP, Aizawa H, Ge P, DiFiglia M, McKee AC, MacDonald M (1995). An improved approach to prepare human brains for research. J Neuropathol Exp Neurol.

[CR24] Vonsattel JP, Del Amaya MP, Keller CE (2008). Twenty-first century brain banking. Processing brains for research: the Columbia University methods. Acta Neuropathol.

[CR25] Waldvogel HJ, Curtis MA, Baer K, Rees MI, Faull RL (2006). Immunohistochemical staining of post-mortem adult human brain sections. Nat Protoc.

